# Rare Invasive Fusarium Sinusitis in a Transiently Immunosuppressed Patient

**DOI:** 10.7759/cureus.107846

**Published:** 2026-04-27

**Authors:** Vincent L Archibald, Senthil R Meenrajan, Denise Schain, Anastasia Tishena, Margaret C Lo

**Affiliations:** 1 Medicine, Duke University Medical Center, Durham, USA; 2 Medicine, University of Florida College of Medicine, Gainesville, USA; 3 Infectious Diseases and Transplant, University of Florida College of Medicine, Gainesville, USA

**Keywords:** antibiotic side effects, antibiotic stewardship, fusarium species, invasive fungal sinusitis, neutropenic fever, transient neutropenia

## Abstract

A Florida cattle rancher undergoing treatment for lumbar osteomyelitis presented with severe neutropenia and febrile episodes after being discharged on a seven-week course of intravenous (IV) vancomycin and cefepime. Upon admission, antibiotics were changed, and micafungin and filgrastim were added. Several days later, he had a normalizing absolute neutrophil count (ANC) and improving fevers with an overall good clinical appearance. Discharge seemed near, but he continued to complain of nasal congestion and mild oromaxillary pain. In caution, a CT maxillofacial scan and ENT evaluation were performed that revealed extensive nasal septal necrosis with cultures growing *Fusarium suttonianum*, prompting treatment with IV liposomal amphotericin, IV voriconazole, and oral terbinafine. Due to reported resistance to FDA-approved antifungals, compassionate use medications were explored. Clinically, he continued to look well and reported minimal symptoms despite the severity of necrosis. The patient responded well to triple antifungal therapy inpatient and continued therapy at home until antifungals were paused due to amphotericin-induced acute kidney injury. He had completed 38/42 days of planned treatment at this point, so the decision was made to hold antifungals (including potential compassionate use medications) until his upcoming ENT debridement. This ENT evaluation then confirmed no residual disease. Continuation of the antifungal regimen and additional compassionate use medications were not pursued, and the patient continued to do well. This case teaches to take the potential hematological complications of many antibiotics seriously and raises further teaching points to consider on the risks and management of rare, rapidly progressing invasive infections such as *Fusarium*.

## Introduction

Fungal infections caused by the genus *Fusarium *represent a critical challenge in the clinical management of immunocompromised patients [1]. As the population of people living with immunosuppression continues to increase, this globally ubiquitous fungus, known historically as a plant pathogen, is causing an increasing number of infections in human hosts through its airborne dispersion [2]. The presentation of *Fusarium* infections is broad, and while typically disseminated, localized disease from *Fusarium *can occur [3-5]. This may lead to life-threatening infections in immunocompromised patients [6]. In our case, the immunocompromised state came from transient severe neutropenia due to antibiotic therapy for osteomyelitis. Neutropenia is a significant adverse effect associated with many antibiotics, including intravenous (IV) vancomycin and cefepime; severity is directly correlated to the duration of therapy [6-9]. 

## Case presentation

A Florida cattle rancher in his 50s with no chronic medical history presented to our quaternary hospital with two months of progressive lower back pain. Magnetic resonance imaging revealed advanced diskitis and vertebral osteomyelitis, accompanied by an epidural abscess at the L1/L2 level. The patient was initiated on IV vancomycin and cefepime and subsequently discharged without any surgical intervention to complete seven additional weeks of antibiotics at home, with weekly lab follow-up. He had no other medication changes made and only took amlodipine prior. Three weeks into antibiotic therapy, the patient’s white blood cell (WBC) count dropped from 8200/µL to 2,000/µL with an absolute neutrophil count (ANC) of 1270/µL. During the following week, he developed febrile episodes (maximum temperature: 39.1°C) that consistently occurred several hours after antibiotic administration. His spouse staggered the administration of the antibiotics to identify the cause of the reaction. The patient continued to experience fevers, chills, and rigors approximately three hours after vancomycin administration, which would resolve within 12 hours. These episodes increased in intensity over time. A repeat complete blood count (CBC) one week later revealed precipitously worsening severe leukopenia, with a manual ANC of zero.

**Figure 1:**
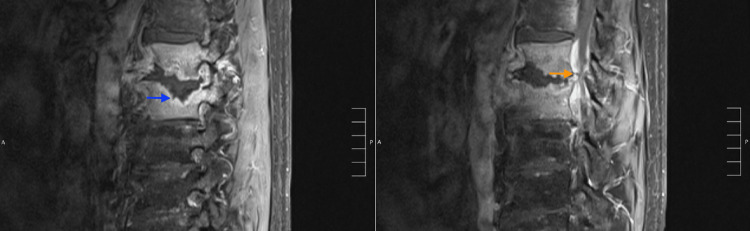
MRI lumbar spine with and without contrast showing findings consistent with advanced discitis, vertebral osteomyelitis, and a compressive epidural abscess at L1/L2 There is erosion of the endplates at L1 and L2 with abnormal edema throughout the vertebral bodies of L1 and L2 and abnormal enhancement within the vertebral bodies. In the disc space there is a rim-enhancing fluid collection and collapsed disc material (blue arrow). At L1 and L2, there is also a circumferential epidural abscess compressing the thecal sac (orange arrow). There is no definite evidence of abnormal enhancement of nerve roots above or below the level of the involvement

Upon his admission for neutropenic fever, both vancomycin and cefepime were discontinued. His regimen was changed to aztreonam and daptomycin for continued treatment of vertebral osteomyelitis and diskitis. Micafungin was added for empiric antifungal coverage given intense febrile episodes with limited data and lack of localizing symptoms. Filgrastim was administered due to profound neutropenia with a relatively unclear timeline. On hospital day 3, both blood and fungal cultures were negative (and remained negative throughout admission). Despite still having severe neutropenia, the patient exhibited no localizing signs or symptoms of infection except for oromaxillary discomfort. Direct examination showed oral leukoplakia, confirmed as *Candida albicans* by culture, and was treated with nystatin swish-and-swallow therapy. On hospital day 4, the patient’s ANC recovered to 400/µL, and febrile episodes resolved. He started to report new nasal congestion and ongoing oromaxillary pain. At baseline, the patient reported seasonal allergies, for which he takes loratadine, and the congestion is no different than his baseline. A subsequent oral exam noted a new erythematous lesion at the site of a missing upper left molar. Given this new finding and persistent ENT complaints in the setting of transient, now resolved, neutropenia, diagnostic evaluation began for concerns of occult infectious pathology.

Investigations

**Figure 2:**
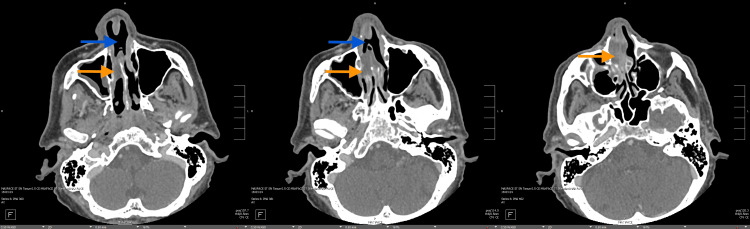
CT maxillofacial without contrast (axial) showed a large nasal septal perforation with mucosal thickening and hyperenhancement along the nasal septum (blue arrow) Evidence can also be seen of an associated lobulated fluid density centered along the nasal septum at the superior and posterior margin of septal perforation extending into the right nasal cavity toward the middle meatus, which was concerning for a nasal septal abscess (orange arrow)

**Figure 3:**
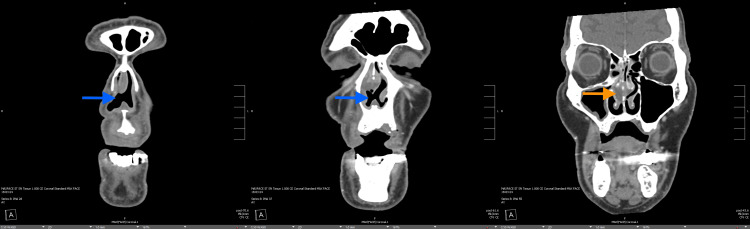
CT maxillofacial without contrast (coronal) concerning for a large nasal septal perforation (blue arrows) and nasal septal abscess (orange arrow) as described in Figure 2

A maxillofacial computed tomography scan on hospital day five demonstrated a large nasal septal perforation with mucosal thickening, hyperenhancement, and associated fluid density concerning for a nasal septal abscess. There were prominent 1B and 2A lymph nodes. There was no evidence of invasive sinusitis, tonsillar abscess, peritonsillar abscess, or tonsillitis at this time (Figures 2-3).

On hospital day 6, ANC normalized (4500/mL) after five total doses of filgrastim. The patient underwent an otolaryngology (ENT) consult. Nasopharyngoscopy inspection of the nasal cavity at the bedside noted extensive necrosis, suggestive of invasive fungal sinusitis. Multiple biopsies from bedside debridement were sent to pathology and showed extensive necrosis with high suspicion for fungal angioinvasion, given findings from Grocott methenamine silver (GMS) and periodic acid-Schiff (PAS) stains, but no definitive diagnosis could be made at that time due to significant necrotic tissue debris. The infectious disease (ID) team empirically switched the micafungin to liposomal amphotericin B (450 mg IV daily in D5W 225 mL).

On hospital day 9, the patient underwent surgical nasal debridement for extensive necrosis with ENT. Intraoperative biopsies were sent to pathology and showed extensive necrosis and angioinvasion in the right middle turbinate, bilateral sinonasal mucosa, right lacrimal duct, and right lacrimal sac, without invasion into the orbit. These biopsies were cultured and identified as 2+ *Fusarium suttonianum *and 2+ *Lomentospora prolificans*. Susceptibilities are noted below (Table 1). Notably, no Clinical & Laboratory Standards Institute (CLSI) interpretation was available for *Fusarium*.

**Table 1:** Sensitivities from initial culture NIA: no interpretative criteria available Concentrations in ug/mL

	Voriconazole	Itraconazole	Amphotericin B	Micafungin	Posaconazole	Isavuconazole	Ibrexafungerp
Fusarium	>16	>16	1	>8	>16	>16	>8
Lomentaspora	NIA	NIA	NIA	NIA	NIA	NIA	NIA

Despite the inherent resistance of *Lomentospora *to amphotericin B in the literature, ID recommended continual therapy with the addition of IV voriconazole 450 mg every 12 hours and oral terbinafine 500 mg every 12 hours for synergy. Both fungal isolates were sent to the Mycology Center at the University of Texas San Antonio (UTSA) for sensitivity testing. A workup was done to look for potential immunodeficiencies or immunocompromising states. The patient's HIV fourth-generation antibody and p24 antigen and Hepatitis C virus (HCV) antibody tests were negative. His immunoglobulin A, G, and M levels were within normal limits. Risk factors that may have predisposed him to these atypical fungi include his occupation, in which he frequently washes his face in trough water. He has no record of maxillofacial surgery, does not use intranasal glucocorticoids, has no recent history of systemic glucocorticoids for his back pain, and has no history of diabetes mellitus.

Treatment

Despite the severity of the pathology findings, the patient remained relatively asymptomatic with mild leukocytosis. The patient’s primary complaint was ongoing nasal congestion, while he comfortably ambulated between antifungal treatments. On hospital day 16, a repeat ENT debridement demonstrated successful resection by ENT to viable tissue in all fields of the nasal cavity. Intraoperative biopsies from this debridement were cultured, and only isolated *Fusarium suttonianum*. It is unclear if *Lomentospora prolificans* was a contaminant, successfully treated, or not captured in the second sample.

Antifungal therapy was continued with liposomal amphotericin B, IV voriconazole, and oral terbinafine. Sensitivity results from UTSA indicated potential susceptibility of *Fusarium suttonianum *to amphotericin B (MIC: 1 ug/mL) and fosmanogepix (MIC: <0.008 ug/mL), while *Lomentospora prolificans* appeared resistant to all FDA-approved drugs but had low minimum inhibitory concentrations to olorofim (MIC: 0.125 ug/mL) and fosmanogepix (0.015 ug/mL). Meanwhile, during this admission, the patient completed the entire seven-week course of antibiotics for his lumbar osteomyelitis, with both radiographic and clinical evidence of treatment success.

ID consult finalized the acquisition of fosmanogepix therapy on an outpatient basis since the patient expressed wishes for discharge, but the patient opted against additional compassionate use medications given his clinical stability. The patient was discharged home in stable condition on hospital day 26 with the antifungal regimen of IV amphotericin, oral terbinafine, and oral voriconazole with documentation of therapeutic voriconazole levels. The plan was to complete 42 total days of triple antifungal therapy for the invasive *Fusarium *fungal sinusitis.

Postdischarge, the patient experienced acute kidney injury (AKI) discovered during weekly lab monitoring, with serum creatinine increasing from a baseline of approximately 1 mg/dL to 1.7 mg/dL, necessitating a reduction in the frequency of amphotericin administration from daily to every other day. Nineteen days after discharge, the patient’s AKI required hospitalization for supportive care, and the antifungal regimen was paused on discharge, having completed all but four days of the planned treatment course. The patient completed 38 days of the triple antifungal therapy. The decision was made to further defer the decision to start fosmanogepix until repeat debridement with ENT, scheduled for outpatient two weeks later.

Outcome and follow-up

At this scheduled ENT follow-up, subsequent nasal debridement showed no further signs of ongoing fungal disease, leading to the mutual decision by ID and the patient to clinically monitor the situation further without ever initiating fosmanogepix. 

## Discussion

This case underscores significant clinical considerations in the diagnostic evaluation of drug-induced neutropenia. It further poses important teaching points on the risk factors for and the management of invasive fungal sinusitis. Firstly, the development of severe neutropenia in our patient illustrates the well-documented risk associated with prolonged antibiotic therapy. One cohort study reported an incidence of neutropenia of 3.9% with vancomycin, and the risk of neutropenia increases with longer periods of treatment, as is true in our case [6]. The likelihood of developing neutropenia increases notably with therapy extending beyond a week, commonly manifesting after 20 days [7]. Similarly, cefepime is well-associated with neutropenia, particularly in prolonged treatments for conditions like osteomyelitis, with occurrences typically noted between 17 and 30 days of therapy [8,9]. This highlights the necessity of close clinical follow-up and regular lab monitoring when employing these agents over long treatment durations.

Additionally, this case illustrates the severe complications that can arise from transient neutropenia, such as rare, life-threatening fungal infections, even in otherwise healthy individuals. Our patient developed invasive fungal sinusitis caused by *Fusarium *species, an occurrence much less common than infections caused by *Aspergillus *or *Mucorales* [10]. *Fusarium *infections typically affect severely immunocompromised patients and are uncommon in clinical presentations confined to the sinuses without disseminated disease [1,3,4]. Our patient did not fit the typical individual at risk for *Fusarium *infection, nor did he display the classic disseminated presentation of *Fusarium *disease. Yet, he did sustain extensive necrosis of the mucosa characteristic of *Fusarium *infection as a result of its angio-invasive spread [4]. His nasal congestion and oromaxillary pain could have been easily dismissed as benign allergic rhinitis or chronic sinusitis, given the transience of his immunocompromised state and overall good clinical appearance. Successful clinical recovery is dependent on the resolution of neutropenia and the extent of the *Fusarium *infection, so the patient’s rapid and favorable outcome with quick normalization of ANC, localized infection, and continual robust immune health during treatment likely contributed to his complete and sustained clinical recovery thus far [5]. With the ubiquity of *Fusarium *in moist environments throughout the world and growing prevalence of infections in humans, his occupational exposure as a rancher, frequently in contact with nonsterile water and soil, may have predisposed him to repeated fungal insult and colonization that ultimately predisposed him to infection during his transient severe neutropenic state [1,2]. Thus, gathering an extensive social history is important and should include travel history, rural living, water, pet, and occupational exposures.

Throughout the treatment course of this patient, repeated questions surfaced about other potential underlying immune dysfunctions that could have predisposed him to his initial osteomyelitis and the rapidly progressing nature of his fungal sinusitis. Given case reports of clinical manifestations of documented *Fusarium *infections directly correlating with the degree and duration of immunosuppression, identification of a pre-existing immune abnormality is important to guide the overall care of *Fusarium *infections, especially when considering compassionate use medications [10,11]. Our patient had none discovered through our investigations. This case teaches a cautionary tale of clinical hypervigilance for potential infectious complications in those at high risk who sustain severe neutropenia, even if transient and otherwise generally healthy. Given its limited treatment susceptibility and poor clinical prognosis, primary prevention against *Fusarium *infections is key to minimizing the risk of both airborne and waterborne exposure [2]. Such preventative measures include avoidance of tap water contact in areas of skin breakdown, strict adherence to air and water precautions with high-efficiency air filters, and thoroughly clean showers [12,13].

## Conclusions

This case discusses a previously healthy man who developed invasive fungal sinusitis with a highly antifungal-resistant organism, *Fusarium*, after developing profound neutropenia 2-3 weeks after starting IV vancomycin/cefepime for vertebral osteomyelitis/discitis. Lessons that may be learned from this case report fall generally into those about antibiotic stewardship, keeping high suspicion for new infection in any immunocompromised individual, and atypical presentations of *Fusarium*.

First, prolonged use of many antibiotics, such as vancomycin and cefepime, is associated with a significant risk of inducing neutropenia, particularly after extended therapy durations of 20+ days. Close clinical follow-up and regular laboratory monitoring are critical when employing high-risk antibiotic agents such as vancomycin and cefepime over long treatment durations. Second, patients with severe neutropenia, regardless of the duration of immunosuppression, are at high risk for serious infectious complications, even in otherwise healthy individuals, and must undergo an aggressive diagnostic investigation for any new or persistent clinical symptoms and/or physical findings. Gathering detailed social history is important to determine additional risks for fungal infections. Finally, while *Fusarium *species infections typically present as invasive disseminated disease and are associated with chronically immunocompromised individuals, *Fusarium *opportunistic infections can occur in transiently immunocompromised patients and may present atypically as localized infections in such individuals.
